# Cumulative assessment: strategic choices to influence students’ study effort

**DOI:** 10.1186/1472-6920-13-172

**Published:** 2013-12-27

**Authors:** Wouter Kerdijk, René A Tio, B Florentine Mulder, Janke Cohen-Schotanus

**Affiliations:** 1 Center for Research and Innovation in Medical Education, University of Groningen and University Medical Center Groningen, Ant. Deusinglaan 1, FC40, 9713, AV Groningen, The Netherlands; 2 Department of Cardiology, University of Groningen and University Medical Center Groningen, Groningen, The Netherlands; 3 Institute for Medical Education, University of Groningen and University Medical Center Groningen, Groningen, The Netherlands

**Keywords:** Summative assessment, Learning effects of assessment, Medical education, Higher education, Knowledge development, Knowledge retention, Test enhanced learning, Cumulative assessment, Repeated testing

## Abstract

**Background:**

It has been asserted that assessment can and should be used to drive students’ learning. In the current study, we present a cumulative assessment program in which test planning, repeated testing and compensation are combined in order to influence study effort. The program is aimed at helping initially low-scoring students improve their performance during a module, without impairing initially high-scoring students’ performance. We used performance as a proxy for study effort and investigated whether the program worked as intended.

**Methods:**

We analysed students’ test scores in two second-year (n = 494 and n = 436) and two third-year modules (n = 383 and n = 345) in which cumulative assessment was applied. We used t-tests to compare the change in test scores of initially low-scoring students with that of initially high-scoring students between the first and second subtest and again between the combined first and second subtest and the third subtest. During the interpretation of the outcomes we took regression to the mean and test difficulty into account.

**Results:**

Between the first and the second subtest in all four modules, the scores of initially low-scoring students increased more than the scores of initially high-scoring students decreased. Between subtests two and three, we found a similar effect in one module, no significant effect in two modules and the opposite effect in another module.

**Conclusion:**

The results between the first two subtests suggest that cumulative assessment may positively influence students’ study effort. The inconsistent outcomes between subtests two and three may be caused by differences in perceived imminence, impact and workload between the third subtest and the first two. Cumulative assessment may serve as an example of how several evidence-based assessment principles can be integrated into a program for the benefit of student learning.

## Background

In medical education, the assertion that assessment drives learning evokes positive and negative reactions [1,2]. Critics state that assessment stimulates learning for assessment rather than learning per se, or that assessment drives surface rather than deep learning [3]. Others are more pragmatic and reason that if assessment drives learning, why not use it to stimulate learning [4]? The common end-of-course test may negatively affect study effort, because students start preparing for a test three to four weeks in advance [5]. Consequently, if a course lasts longer than three to four weeks, students will be less engaged with the content during the first part of the course, which may impair their learning. In this exploratory study, we present a cumulative assessment program which combines frequent testing, repetition of content and compensation among tests in order to stimulate students’ study effort.

In the preclinical phase, medical knowledge is often assessed by written tests. Students’ performance on written tests can be influenced by their study effort [6], which, in turn, can be influenced by characteristics of the assessment program. Test dates and deadlines determine when students spend time on test preparation and other academic tasks [5,7]. Instead of studying from the beginning of a course, students tend to start studying when the test date comes closer, which is called academic procrastination [7]. It is estimated that 95% of students procrastinate to some extent and up to 30% procrastinate to such an extent that they delay many of their tasks until just before or even beyond the deadline [8-10]. Students, on average, start preparing for a test three to four weeks in advance [5]. Consequently, regular tests every three to four weeks should support students to put continuous effort into their learning.

Repeated testing also encourages students to put effort into studying the same content repeatedly. Repetition of content has been demonstrated to improve retention [11,12]. People learn and retain information better through repeated exposure [13]. Actively retrieving content during a test strengthens retention even more [11,14]. Consequently, for an assessment program to be effective, the same content should be repeatedly tested and assessment within a course should be organized in such a way that each test includes the study material from preceding tests.

When using multiple tests to assess the same content, it is advisable to combine test scores and allow for compensation between the tests within the course. Compensatory assessment enables students to compensate poor performance on one test with good performance on others [5,15]. A major advantage of compensatory assessment is that students are not discouraged too much by initial poor test results, since there is still a possibility for repair, which encourages increased study effort. A possible disadvantage of compensatory assessment is that initially high-scoring students might refrain from studying intensively for the next test. However, if each subsequent test has an increasing number of items, initial good test results will not guarantee a successful final grade. This way, all students will have to keep studying to pass the entire assessment program. For a compensatory assessment program to be effective, a condition is that students receive information about their performance between the tests. This information should help students correct their errors and reinforce correct responses [16-18]. It should not be provided during a test or when other activities require students’ attention, but rather when students are in a position to actively process it [18,19].

The cumulative assessment program under study is designed to encourage students to continuously study throughout a course. We expect students with an initial low test score to benefit from the program, because it offers them the opportunity to identify knowledge deficits and compensate initial poor performance with higher performance on subsequent tests. Frequent and repeated testing offers students the opportunity to repeatedly recall the course content and remedy their knowledge deficits. The cumulative assessment program can be expected to be less beneficial for students who scored high on the first test, since there is less room for improvement. However, frequent testing with an increasing number of questions and weight per test should stimulate high-performing students to keep putting effort into studying. Repetition of content should increase their retention as well and help them maintain their high scores. In summary, we expect the cumulative assessment program to benefit the performance of initially low-scoring students, without impairing that of initially high-scoring students. Therefore, we expected initially low-scoring students to improve their scores on subsequent tests and initially high-scoring students to retain relatively high scores.

## Methods

### Context

The undergraduate medical curriculum of the University of Groningen comprises a three-year preclinical bachelor’s program and a three-year clinical master’s program. Cumulative assessment is implemented throughout the bachelor’s program.

The cumulative assessment program is applied to ten-week modules in which different content areas are integrated. All content of a module is assessed by one multiple choice test. The test is divided into three separate mandatory subtests scheduled at the end of weeks four, eight and ten of the module (*frequent testing*). Each subtest contains questions covering the content of all preceding weeks (*repetition*). The final grade is based on the total number of questions from the three subtests, and is calculated at the end of a module (*compensation*). Shortly after each subtest, information about students’ performance is provided through the digital learning environment by publishing the correct answers and the number of questions each student answered correctly.

The distribution of the content of a module over three subtests is based on a conceptual model, in which the content of each week is assessed using the same number of multiple choice questions. Each subtest contains an increasing number of questions, covering the content of all preceding weeks. In Table 1 this model is specified for a test of 200 questions, covering each week with 20 questions. The first subtest contains 50% of the questions regarding the content of the first four weeks. The second subtest contains 25% of the questions about the content of the first four weeks and 50% of the questions about the content of weeks five through eight. The final subtest contains the remaining questions: 25% of the questions about the content of the first four weeks and 50% of the questions about the content of weeks five through eight, and all questions about the content of the last two weeks. This distribution of questions over subtests results in an assessment program in which students can compensate for low initial scores, without making one of the subtests superfluous for initially high-scoring students.

**Table 1:** Conceptual model of a 10-week cumulative assessment program

**Week**	**1**	**2**	**3**	**4**	**5**	**6**	**7**	**8**	**9**	**10**	**Total**
Subtest 1	10	10	10	10							40
Subtest 2	5	5	5	5	10	10	10	10			60
Subtest 3	5	5	5	5	10	10	10	10	20	20	100
total row	20	20	20	20	20	20	20	20	20	20	200

Conceptual distribution of questions over three subtests in a 10 week cumulative assessment program, each week being assessed with 20 questions.

### Participants

We used students’ test results from two second-year modules: modules 1 and 2 (n = 494 and n = 436, respectively) and two third-year modules: modules 3 and 4 (n = 383 and n = 345, respectively). The data were gathered during the time that, under Dutch law, educational studies were exempt from institutional board review. In accordance with the university privacy policy and Dutch Law, data were derived from the student records and anonymized before analysis.

### Analysis

**Figure 1:**
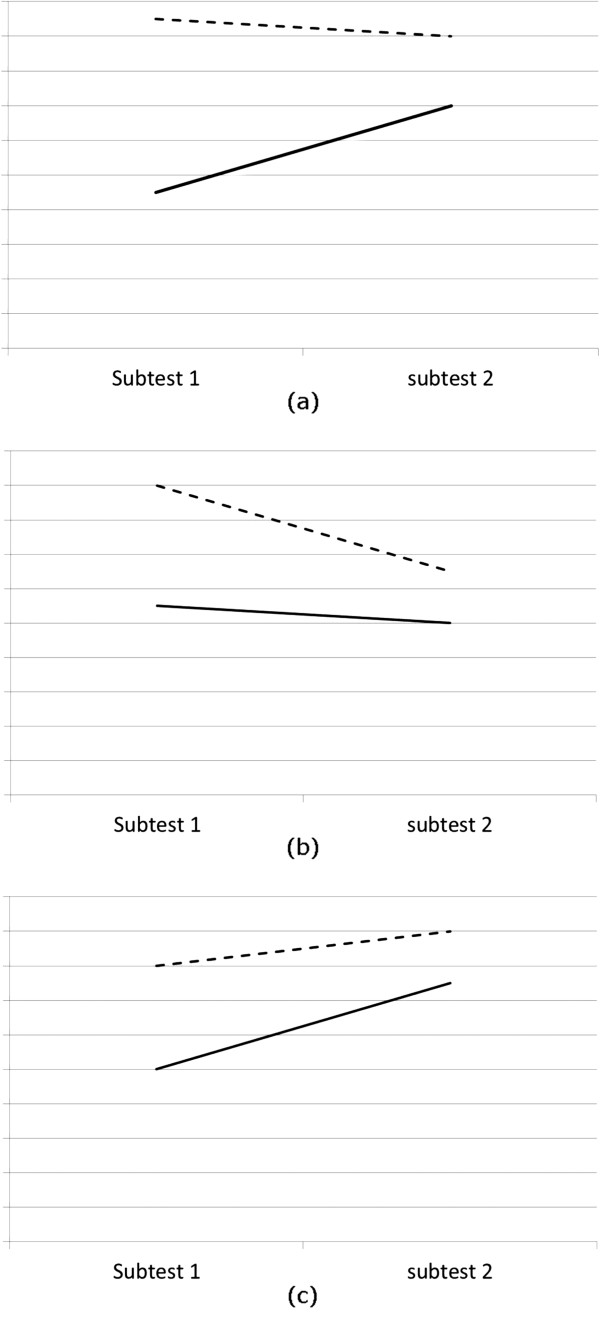
**Expected score change for different test difficulties.** Directions of expected score change for initially low-scoring students (solid line) and initially high scoring students (dashed line) when the second test is equally difficult **(a)**, more difficult **(b)** or less difficult **(c)** compared to the previous test.

To test our expectations we compared the score change between tests of initially high and low-scoring students as a proxy for an increase or decrease in study effort. During the analysis we faced two challenges. First, we had to take into account regression to the mean. Regression to the mean is caused by random measurement error when the same participants are repeatedly measured [20]. Based on this statistical phenomenon, one would expect the high-scoring group to have a lower score and the low-scoring group to have a higher score on a subsequent test, purely due to personal variation. To ensure that the results of our study were not caused by regression to the mean, we judged cumulative testing beneficial when the mean difference in test scores between two tests was larger for low-scoring than for high-scoring students (Figure 1a). When the direction of the mean difference of one group was positive and that of the other group negative, we compared the absolute mean differences.

Our second challenge was that, when comparing students’ performance on two different tests, differences in test difficulty might systematically bias the results. In our medical school, knowledge test items are teacher-made and checked in-house on face validity by a peer and an educationalist. Therefore, there was no a priori knowledge about the difficulty of the subtests available. Consequently, subtest difficulty could not be controlled and could vary substantially. All students in a module took the same tests, so low and high-scoring students’ test scores should have been affected by test difficulty in the same way. However, during the interpretation of the comparisons between high and low-scoring students’ score change, we needed to take test difficulty into account because it may change the direction of the mean score change between two tests for one of the groups. If the second subtest is more difficult than the first one, we would expect both groups to decrease in score. If cumulative assessment has an effect, we would expect high-scoring students’ scores to decrease more than those of low-scoring students (Figure 1b). Similarly, if the second subtest is less difficult than the first one, we would expect an increase in scores of both groups and the low-scoring students to improve more, due to cumulative assessment (Figure 1c). We operationalized test difficulty as the average facility index of the items of the test – the proportion of students that sat the test that answered the question correctly.

To enable comparison between subtests, we calculated the percentage of correctly answered questions for each subtest. Subsequently, we identified low and high-performing students by selecting the lowest and highest quartile, based on students’ performance on the first subtest. We used independent sample t -tests to compare the mean differences of the low and high-performing groups between subtests 1 and 2.

We expected students to revaluate their performance and adjust their study behaviour after they received new information about subtest 2. Therefore, we identified new quartiles of low and high-performing students after subtest 2, based on the combined score on the first two subtests. Again, we used independent sample t-tests to compare the mean differences of the low and high-performing students between the combined subtests 1 and 2, and subtest 3.

## Results

For each of the four modules, the difficulty level of each subtest is reported in Table 2.

**Table 2:** Difficulty per subtest and module

**Module**	**Difficulty Subtest 1 (p)**	**Difficulty Subtest 2 (p)**	**Difficulty Subtest 3 (p)**
1	0.65	0.68	0.66
2	0.68	0.62	0.67
3	0.68	0.68	0.74
4	0.68	0.73	0.75

Difficulty per subtest per module expressed as the average proportion of questions answered correctly by all students who sat the subtests.

Comparing the mean differences between subtests 1 and 2 of initially low and high-scoring students, we found significant differences in score change for all four modules. In modules 1, 3 and 4 the difficulty of the second subtest was only slightly higher than that of the first one. In these modules, we found the average improvement of low-scoring students to be significantly higher than the average decrease in high-scoring students’ scores, which is in line with our expectations (Table 3). In module 2, both groups decreased in scores as expected based on the higher difficulty of subtest 2. On average, high-scoring students scores’ decreased significantly more than low-scoring students’ scores.

**Table 3:** Results comparing subtests 1 versus 2

**Module**	**Group**	**Students**	**T**_**1**_	**T**_**2**_	**Absolute difference**	***T*-test**	
		**n**	**Mean**	**Mean**	**|Δ|**	**t**	**p**
1	Initial low scorers	111	49.82	59.36	|9.54|	8.19	.00*
	Initial high scorers	139	73.29	72.27	|-1.02|		
2	Initial low scorers	100	53.17	52.12	|-1.05|	6.49	.00*
	Initial high scorers	122	77.21	68.29	|-8.92|		
3	Initial low scorers	82	50.78	59.01	|8.24|	2.52	.01*
	Initial high scorers	109	71.58	65.71	|-5.87|		
4	Initial low scorers	96	43.28	52.10	|8.82|	9.66	.00*
	Initial high scorers	76	58.90	57.77	|-1.13|		

For initial low and high scorers in four modules: mean test scores and absolute difference and t-tests comparing their absolute mean difference in test scores between subtests 1 (T_1_) and 2 (T_2_).

* = significant at the α = 0.05 level.

When we compared the mean difference between the combined subtests 1 and 2, and subtest 3, we found significant differences in modules 1 and 3 (Table 4). In module 1, where test difficulty was similar between tests, the scores of low-scoring students increased whereas those of high-scoring students’ decreased. Contrary to our expectations, the decrease in scores was significantly higher in the high-scoring group than the small increase in scores in the low-scoring group. In module 3, the third subtest was less difficult than subtests 1 and 2. Therefore, both groups showed improvement between the first two and the third subtests. In line with our expectations, the scores of the low-scoring students increased significantly more than those of high-scoring students. Against expectation, we found no significant differences in score change between subtests 2 and 3 in modules 2 and 4.

**Table 4:** Results comparing subtests 1 and 2 versus 3

**Module**	**Group**	**Students**	**T**_**1+2**_	**T**_**3**_	**Absolute difference**	***T*-test**	
		**n**	**Mean**	**Mean**	**|Δ|**	**t**	**p**
1	Initial low scorers	133	54.30	55.19	|0.89|	−3.24	.00*
	Initial high scorers	124	75.04	71.16	|-3.88|		
2	Initial low scorers	107	51.05	53.50	|2.45|	.97	.33
	Initial high scorers	110	74.02	75.57	|1.54|		
3	Initial low scorers	106	55.08	62.23	|7.16|	7.00	.00*
	Initial high scorers	101	69.56	71.15	|1.58|		
4	Initial low scorers	87	46.71	69.04	|22.32|	-.02	.98
	Initial high scorers	83	58.93	81.27	|22.35|		

For initial low and high scorers in four modules: mean test scores and absolute difference and t-tests comparing their absolute mean difference in test scores between the combined subtests 1 and 2 (T_1+2_) and subtest 3 (T_3_).

* = significant at the α = 0.05 level.

## Discussion

In this study, we presented a cumulative assessment program that is strategically designed to influence student learning. We found evidence for our expectation that initially low-scoring students will improve their scores on subsequent tests while high-scoring students will retain a relatively high score. The effect was most obvious between the first and the second subtests. Between subtests 1 and 2, the scores of initially low-scoring students increased significantly more or decreased significantly less than the scores of initially high-scoring students decreased. Taking into account the difficulty of each subtest, we found support for our expectation in each module. Our finding suggests that our cumulative assessment program encourages low-scoring students to increase their study effort, while it stimulates high-scoring students to keep up their study effort.

The underlying assumption of our study is that students’ changes in test scores reflect their study effort. In the literature, test performance has also been linked to other factors such as learning strategies and deep learning [21-24]. However, effective deep learning is associated with study effort and applying different learning strategies requires students to put in effort as well [21]. Furthermore, a recent study has shown that the positive effect of factors such as deep learning and resource management on student performance is mediated by student participation, which is a form of study effort as well [24]. Further research should establish whether our results can indeed be attributed to an increase in study effort and whether cumulative assessment leads to more participation or other changes in study strategies.

The results between subtests 2 and 3 were less clear. We only found a significant difference in two out of four modules. The results for module 3 confirmed our expectation that initially low-scoring students would improve more than initially high-scoring students. The results for module 1 revealed that the scores of initially high-scoring students decreased more than the scores of low-scoring students increased. We did not find a significant difference in the other two modules. These varying findings may have been caused by general effects of assessment on learning behaviour. Recently, Cilliers et al. found that the imminence of assessment, the perceived impact of the test and the amount of workload associated with the test generally affect the way students learn for their exams [25,26]. In our cumulative assessment program, compared to the first two subtests, the third subtest determines 50% of the final grade and covers the content of the entire module. Besides, there are only two weeks between subtests 2 and 3. One could imagine how students may perceive the third subtest differently than the first two, when it comes to imminence, impact and workload of assessment. Furthermore, with only two weeks left before the next test, students may not have been able to adjust their study effort after evaluating their deficits. We argue that these factors may have affected students’ learning behaviour more during their preparation for the third subtest than for the other two subtests. Perhaps, an increase in imminence, impact and workload of subtests may influence students’ performance and study behaviour more than the cumulative assessment program.

Our cumulative assessment program is well-grounded in theory and combines frequent testing, repetition of content and compensation among tests [5,12,15,19,27]. Several studies report positive effects of repeated testing of content in isolated courses [12,28-30]. In these studies, tests were added to the regular program of a single course and were not part of a formal assessment program. The beneficial effects of the other two aspects of our cumulative assessment program have mostly been established in laboratory studies and simulated classroom experiments. This study adds to the literature by investigating these principles in a naturalistic setting. Furthermore, our study was embedded in a formal assessment program, which raises the stakes for students and causes an increased ecological validity of our findings. However, our findings are limited to the extent that we cannot attribute them to any separate aspect of the program. Further research is necessary to understand the interplay and separate roles of these aspects in the cumulative assessment program.

The use of naturalistic data, has other possible limitations. Both the student sample and the characteristics of modules and tests can be seen as potential sources of bias. To minimize the influence of such bias, we investigated four modules to see whether the results were the same for different modules. Furthermore, during the interpretation of our results we took regression to the mean and test difficulty into account. Indeed, any difference in test difficulty between two tests or between modules was the same for all students, which increased the validity of our outcomes.

The findings in this exploratory study about the effects of a cumulative assessment program seem promising and add to the evidence that assessment can be used to support student learning. We cannot be sure whether cumulative assessment stimulates deep learning or other beneficial learning behaviours. However, in over half of the tests, initially low-scoring students increased their performance, while initially high-scoring students did not equally decrease in their performance. This suggests that implementing a cumulative assessment program may benefit students’ study effort and test performance. To support this evidence, an experimental design in a high stakes setting could help to further establish the value of cumulative assessment for educational practice.

## Conclusion

The cumulative assessment program under study seems to influence study effort positively. How its influence may be mediated or moderated by the perceived imminence, impact and workload of the test requires further investigation. Based on our findings, we argue that implementing a cumulative assessment program may benefit students’ study progress. Furthermore, we feel that cumulative assessment serves as a good example of how several evidence-based principles of assessment can be integrated into a program that benefits students’ learning.

## Competing interests

The authors declare that they have no competing interests.

## Authors’ contributions

The cumulative assessment program was designed by JCS. All authors were involved in the conception and design of this study. RT and FM gathered the data. WK analyzed the data. All authors interpreted the data together and were involved in drafting and revising the manuscript. All approved the final manuscript.

## Authors’ information

Wouter Kerdijk, Msc, is a psychologist and researcher in Medical Education at the Center for Research and Innovation in Medical Education, University of Groningen and University Medical Center Groningen, Groningen, The Netherlands.

René A. Tio, MD, PhD, cardiologist is associate professor at the department of cardiology and chair of the joint examination committee of Medicine and Dentistry, University of Groningen and University Medical Center Groningen, Groningen, The Netherlands.

Florentine (B. F.) Mulder, Msc, is a psychologist and researcher in Medical Education at the Institute for Medical Education, University of Groningen and University Medical Center Groningen, Groningen, The Netherlands.

Janke Cohen-Schotanus, PhD, is professor in Research in Medical Education and Head of the Center for Research and Innovation in Medical Education, University of Groningen and University Medical Center Groningen, The Netherlands.

## Pre-publication history

The pre-publication history for this paper can be accessed here:

http://www.biomedcentral.com/1472-6920/13/172/prepub
